# 

**Published:** 1876-04

**Authors:** 


### Contents of April Number.

### ORIGINAL.

ART. I.—Clinical Conversations on Diseases of the Skin. By L. Duncan Bulkley, M. D., ........................................ 321
ART. II.—The Sympathetic Nervous System. By S. W. Wetmore, M. D.,. 328
ART. III.—Exsection of the Femur for gun-shot injury. Five inches of bone removed. By Conrad Diehl, M. D., ........................ 342
ART. IV.—Transactions of the Buffalo Medical Association, February 1st, 1876, .................................................. 343

### MISCELLANEOUS.

Ovarian Cyst, Ovariotomy, Menstruation from the pedicle, ................ 350

### EDITORIAL DEPARTMENT.

Foreign Quacks and American Medical Colleges, ........................ 353
Homœopathy and the Michigan University ............................ 355

Books Reviewed:

A Treatise on the Diseases of Infancy and Childhood. By J. Lewis Smith, M. D., .................................................. 357
The Archives of Dermatology, ...................................... 357
Extra-Uterine Pregnancy. By John S. Parry, M. D., ............... 358

Books and Pamphlets Received, ........................................ 360
New Publications ..................................................... 360

THE BUFFALO

